# Five Myths about the HIV Epidemic in Asia

**DOI:** 10.1371/journal.pmed.0030426

**Published:** 2006-10-03

**Authors:** Peter Godwin, Nigel O'Farrell, Knut Fylkesnes, Sujaya Misra

## Abstract

Godwin and colleagues say that there are several myths about the HIV epidemic in Asia, myths that are disseminated in both public and professional discourse and that often dominate policy and political debate.

It is widely recognised that the huge population sizes of many Asian countries mean that although national HIV prevalence levels are still very low, very large absolute numbers of people are being infected each year with HIV [1]. Urgent responses are required; the effective responses by countries such as Thailand and Cambodia have shown how much can be done.

As implementers who have worked with HIV/AIDS programmes in several countries in the region, we recognise the public health and welfare costs of the epidemic in Asia, and we respond to the need to “act now”. We are concerned, however, about a number of misinformed beliefs, or myths, about the epidemic—myths that are widely circulating in Asia, disseminated in both public and professional discourse, and often dominating policy and political debate. We believe that these myths, if allowed to underpin and influence policy and programming and guide immediate action, have the potential to seriously jeopardise exactly the kind of focused, coherent, evidence-based programme being called for in Asia and the Pacific.

In this Essay, we set out five myths that are commonly held with regard to HIV in Asia. We also suggest areas of policy that require greater clarity.

## The Five Myths

**Myth one: There is a major risk that the epidemic in many Asian countries will have the same disastrous “development impact” as in sub-Saharan Africa, but on a much worse scale, given the huge population sizes of much of Asia.** The Asian-Pacific epidemics are very different to those in Africa. The former are concentrated in identifiable high-risk situations (primarily those involving sex workers and injecting drug users [IDUs] who share needles). Hence HIV in the Asia-Pacific region could be controlled if these high-risk situations were targeted with specific interventions [2,3].

We believe that the epidemics in Asia will not become “generalised”, because women's sexual risk is curtailed by social and cultural factors. Age of sexual debut, age of marriage (see Table 1 and [4]), and number of lifetime partners are all very different in Asia compared with Africa, significantly limiting women's (and men's) sexual risk.

**Table 1:**
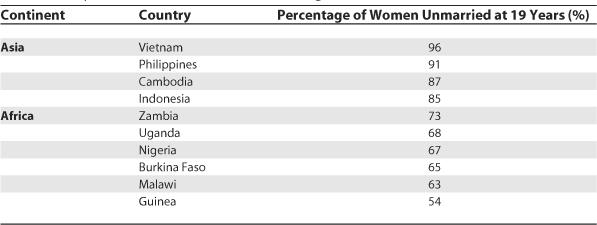
Proportion of Women Unmarried at Age 19 Years (Data from [4])

Many of the Asian epidemics, as mentioned, are currently driven by needle-sharing among IDUs [5]. While IDUs who share needles tend to infect their partners, there is so far little compelling evidence to suggest significant epidemic spread from the drug-using community to those outside this community, even when the IDUs or their partners are sex workers. So far it appears that the kinds of sex work situations which lead to major epidemics (e.g. in Bangkok and Chiang Mai, Thailand, in Mumbai, India, and in Cambodia) do not coincide with major IDU needle-sharing networks. It remains to be seen to what extent the overlap of sex work and drug-use networks, which appear to be occurring in relatively isolated situations in areas such as Ho Chi Minh City, parts of Myanmar, and Yunnan, have the potential to drive major epidemics among the general population.

What is clear, however, is that serious epidemics are occurring among the “high-risk groups” in a number of countries. And while this undoubtedly presents a serious public health problem for the region, it is a problem that is unlikely to have a major developmental impact [6]. Its main impact will rather be in presenting countries with particularly difficult problems of ensuring effective, equitable services, both for prevention and for care, for a series of generally marginalised populations [7]. Many countries in Asia face difficult public policy and legislative problems with regard to sex work, homosexuality, and drug use. In addition, widespread poverty, and a general lack of access to effective health and welfare services by the poor and disadvantaged in both rural and urban areas, means that the challenges of developing targeted intervention programmes, and ensuring coverage of vulnerable groups, are particularly acute. Attention to these challenges, rather than the mythical generalised developmental challenges, is urgently required.

**Myth two: The “Three Ones” are an essential framework for an expanded and strengthened response.** The concept of the “Three Ones”, a strategy to better coordinate the scale-up of national AIDS responses, (http://www.unaids.org/en/Coordination/Initiatives/three_ones.asp), is misleadingly attractive and simple: one national coordinating authority, one strategic plan, and one monitoring and evaluation system. But the idea that such a framework is relevant to Asia is a troubling myth.

Two of the largest global donors for HIV/AIDS, the World Bank and the United Kingdom Department for International Development, though both officially committed to the “Three Ones”, have recently expressed certain reservations about the strategy. The World Bank's evaluation of its US$ 1 billion Multi-Country AIDS Program, [8] and a recent review for the United Kingdom Department for International Development [9], caution that simply establishing national coordinating bodies may often create more problems than it solves—involving, as it often does, attempts to foist additional layers of government upon the implementation of programmes.

Responsibility to coordinate, without the authority to control and ensure coordination, is meaningless. The UNAIDS survey of the “Three Ones” recognised this dilemma: while 80 percent of countries had “national AIDS authorities” who were “recognized as the main coordinator” “with a clear mandate to coordinate”, only 41 percent had “authority to allocate resources” [10]. AIDS authorities need technical and professional support for decisions about resource allocation, priorities, and technical policies. But many national coordinating authorities lack expertise in the specific health, education, rural development, social welfare, or other “development” impacts of HIV. As a result they easily become hostage to political considerations and squabbles over territory and resources.

Sectoral ministries, such as health, education, or social welfare, have the responsibility and authority to develop and implement policies, strategies, and plans in their relevant sector to respond to social, economic, and environmental changes. Yet in many Asian countries these sectoral plans are often weak or lacking with respect to HIV/AIDS. Effective development of such sectoral plans would be far more useful than one national strategic plan—which in any event should, rightly, be a composite of sectoral plans [11]. The myth of “Three Ones” suggests that one national coordinating body is essential to ensure that this multi-sectoral response is developed. But multisectoralism should not be seen as one specific “multi-sectoral strategy”—something operating outside sectors. Instead, multisectoralism simply means the development of strategies in multiple sectors, each one addressing the epidemic and its effect. Attempts to establish or support “one” national institutional coordinating mechanism, with one plan, may therefore be misguided [12,13].

In Asia there is now a growing recognition that while the “Three Ones” may be seen to be important for donors, they are largely irrelevant for countries themselves. What is important for countries is “ownership”—strong leadership, with vision and capability, in government programmes that make maximum use of the contributions of other partners and stakeholders [14].

**Myth three: Most of the progress made in controlling the epidemic in Asia has been made by nongovernmental organisations; the governmental contribution has been limited, clumsy, and hesitating.** Nongovernmental organisations (NGOs) have indeed played a major role in developing innovative approaches and conducting much of the initial ground-breaking progress in the region—but their reach has generally been limited. In many countries, the vulnerability, isolation, and stigmatisation of the target groups arise largely from behaviours which are socially and legally unacceptable within these countries. Paradoxically, only governments can really work effectively, on the scale required, with such groups. Where there is serious commitment, at least officially, to enforcing laws against drug use, prostitution, or illegal migrants, for example, NGOs who try to work with such groups will face harassment and intimidation, if not outright penalties.

Governments can, and often do, choose to allow NGOs to work with “high-risk groups”. This helps governments to achieve their public health goals without appearing to endorse high-risk behaviours (such endorsement could risk losing the support of voters or party members). The opaque nature of such implicit but unacknowledged government backing and support generally limits what can be achieved by the NGOs. The work of NGOs is almost always on a very small scale, with limited coverage. Where governments choose to be pragmatic about the legality of high-risk behaviours, and work directly with high-risk groups, or in explicit partnership with NGOs, much larger-scale coverage can be achieved. Good examples have been the 100 percent condom use programmes with sex workers in brothels in Thailand and Cambodia, and the harm reduction programmes starting with IDUs in Vietnam and China.

A challenge facing HIV/AIDS policy and strategy in the region is a growing awareness of the connections between governance, corruption, social exclusion, economic growth, and the inherent problems caused by the vast concentration of poverty in the region. These issues are critical for HIV policy since, in much of the region, HIV is presently concentrated among the marginalised and socially excluded. There is growing awareness, too, that these will be critical issues as access to treatment expands. Lifetime supplies of HIV medicines will be required, as will high-quality health-care provision. The policy challenge will be not so much the supply of drugs, their prices on the open market, or the costs to patients, but rather in the continuing and consistent procurement and distribution of very large amounts of expensive drugs, and the distortions this may create in under-funded health systems and an under-regulated private sector [15]. Despite their wishes to be more involved, NGOs will in general find only marginal roles to play in addressing these issues: these are the major policy issues governments have to deal with.

Thus while NGOs may have a role as innovators, as watchdogs for human rights, and as advocates for more progressive policies on behaviour change, it is the public sector that has the primary responsibility, and capability, for establishing policy, regulation, accountability, strategy and, by and large, the bulk of service delivery [16]. It is thus essential to recognise the importance of public sector institutions, the roles they have to play, and the importance of strengthening them. There are specific situations, in a very few countries, where governments are doing close to nothing to protect their people, either through gross incompetence or mere neglect. In these situations, NGOs currently do provide the only alternative. But these are specific and special situations, which are not widely generalised.

**Myth four: The Global Fund to Fight AIDS, Tuberculosis and Malaria has recently made a very significant contribution towards controlling the epidemic by making large amounts of funding easily available.** The one undeniable fact about the monies from the Global Fund so far is that they are very difficult to use. There seem to be several reasons for this. First, although the Fund was launched as a “clean, agenda-less, simple-to-use fund”, it does have an agenda. Just as donor countries have (political) agendas that guide their aid and support, so has the Global Fund. The fund's agenda is about building partnerships, involving civil society and those affected directly by the diseases it deals with in the response, and achieving visible, immediate, measurable results [17].

Regardless of how acceptable or otherwise this agenda is to various groups and countries, it is an undeniable “agenda”. And it is new, and very few countries have the institutional bases that can respond to it. In much of Asia, countries tend not to plan and manage in partnership with “civil society”. In some, civil society, as recognised in the West, barely exists formally: the distinction between non-political and political association is essentially not recognised. In some countries the so-called “mass movements”, such as youth and women's unions, are referred to as NGOs; other associations and organisations struggle for recognition and acceptance. Even where NGOs flourish, few country strategies seriously allocate roles, responsibilities, and resources for partnerships with them, or really support, or even allow for, the kinds of partnerships the fund calls for.

To deal with the Global Fund, therefore, countries have had to set up new mechanisms—the Country Coordinating Mechanisms. These, being new and not yet institutionalised, are fraught with problems [18]: they are not the way governments or countries in the region normally manage programmes [19]. The primary effect of these new mechanisms has thus been to significantly raise transaction costs, and duplicate planning, coordination, and reporting systems, while increasing the opportunities for mismanagement and poor governance—if not to jeopardise the possibility of receiving funding at all.

Second, the Global Fund is committed to a risky strategy, at least in Asia. It aims to make large amounts of money available, *in addition* to what is already being used. But many programmes do not have the capacity to suddenly scale up and absorb very large amounts of additional money, and use it all well and quickly [20]—the Fund money has therefore either moved only very slowly, or may have simply replaced other donor funding [21]. This is not the way development works—and HIV/AIDS programmes are very much in the process of development. Large programmes are almost always the result of extended, patient, dialogue to establish what the real needs are, and what institutional capacity there is to absorb them. But the Global Fund's timetable and requirements for the various rounds of funding have tended to bypass good strategic planning and careful analysis of need. And because none of the mechanisms are institutionalised, the Fund tends to push countries into ad hoc projects and vertical (disease-specific) programming—which runs counter to the efforts of countries that are trying to develop comprehensive, coherent strategies and management systems [22,23].

An associated problem is the emphasis on immediate demonstrable results. Not only are there often not the institutional bases to deliver these results immediately, there is often not even the institutional basis to measure them—which can lead to a “trivialising” of indicators of success. To achieve a series of short-term goals that happen to be very expensive, the Global Fund's approach might be valid. But for the long-term task of building health and social welfare systems to produce “Universal Access” [24], this approach is perhaps naïve.

**Myth five: The “expanded multisectoral response”, beyond the health sector, is essential for effective control of the epidemic in Asia.** The vulnerable populations that need coverage are, by their nature, largely discriminated against and isolated from the general services which could reduce their vulnerability: education, welfare services, employment, etc. But in many countries it has now been shown: that provision of specific health-care services for the vulnerable and marginalised can be relatively easily achieved; that such services are cost-effective [25]; and that these are critical in reducing transmission risk. The most obvious of these services are: treating and preventing sexually transmitted infections in sex workers (through mobile clinics, 100 percent condom use programmes, or periodic presumptive treatment); condom distribution programmes (socially marketed or freely distributed); harm reduction (methadone substitution and needle exchange); and peer education and outreach.

The strong links developing between the opportunities offered by, and resources available for, access to treatment and care and targeted prevention programmes, have emphasised this comparative advantage for the health sector. But while the health sector itself has recognised for years that good public health has always had a multi-sectoral aspect, and that effective programmes always work with the cooperation of local authorities and other sectoral collaboration, the territorialities, particularly of United Nations agencies, continue to create confusion, duplication, competition and waste under the name of “the expanded multi-sectoral response”. The emphasis on multi-sectorality may be appropriate in situations where prevalence rates are so high as to seriously affect labour productivity, availability of human resources, and social infrastructure and institutions (as in parts of Africa); but nowhere in the Asia-Pacific region is prevalence so high, or likely to become so high [26,27].

Asian countries have been making significant progress in recent years in providing comprehensive health care to their populations, especially in addressing the challenge of services targeted at the poor, the isolated, and the marginalised. The additional burden to health-care systems posed by even relatively low levels of HIV prevalence among such groups presents a serious long-term threat. Such a threat far outweighs the likelihood of possible serious “multisectoral” socioeconomic devastation. HIV programmes need to recognise this threat, and respond to it urgently.

## Conclusion

There is no doubt that HIV/AIDS is a significant public health problem in Asia and the Pacific. And although virtually all countries have established national and provincial organisational structures to develop a response to HIV/AIDS, these organizations require further strengthening. Perhaps the biggest challenge is lack of organisational and institutional capability to deliver effective prevention and care services at grass-roots level. Yet to be effective, the response to this challenge must be based on good evidence of each country's specific epidemiological needs, proven and working mechanisms for developing programmes and channeling funds, and frankness, openness, and clarity of purpose and process. Building responses to the challenges based on myths about what works, what the situation is, and what is needed, will, however, only bring frustration and heartbreak and perpetuate the suffering of those affected.

## 

**Figure:**
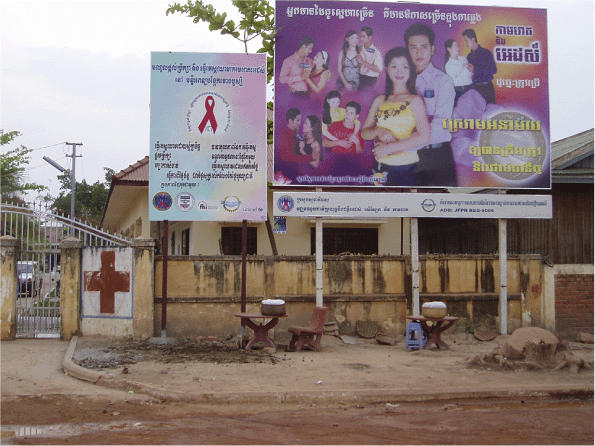
Billboards at Maung Russey Hospital, Battambang, Cambodia, advertising voluntary and confidential counselling and testing services and condom promotion

## Abbreviations

IDU: injecting drug user
NGO: nongovernmental organisation
